# The Wind of Change in Psychiatric Publications

**DOI:** 10.4306/pi.2008.5.2.67

**Published:** 2008-06-30

**Authors:** Peter Tyrer

**Affiliations:** Department of Psychological Medicine, Imperial College, London, UK.

**Keywords:** Bibliometrics, Impact factor, Publication, Psychiatric research

## Abstract

It is getting more difficult to get papers published that it used to be, even though many more journals are available. This article is written mainly for young researchers who are ambitious to get their research published in the best possible journals. A systematic strategic policy is suggested that does not necessarily contradict the aims of achieving the best possible science.

## Introduction

Many years ago one of my educational colleagues carried out a survey into medical students' attitude towards their examinations. This allowed all the students to be divided into three groups, the 'last minute', 'deep thinking' and 'strategic' groups. The 'last minute' group regarded their time at medical school as one of 'personal development', or, in other words, an opportunity to have a good time, and right at the end of their medical school careers realised there was an examination which required some learning. They therefore went into purdah for about four weeks before their examinations, meeting together in huddled groups and learned as much as possible as quickly as possible in the shortest possible time. The hope was, and it was usually fulfilled, that this exercise in short term memory would enable them to cross the hurdle between totally ignored student to highly respected doctor. The 'deep thinking' group were the ones praised and admired by their teachers. They not only attended lectures diligently but asked intelligent questions and desired additional literature on each subject. As a consequence they became so well grounded in the subjects of the teaching that they could echo their teachers and help their fellow students. Although this group was the most desired by the medical school they were the least in number.

The third group, which constituted the majority, were strategic in their attitude towards examinations. They worked out well in advance which subjects were likely to come up in examinations, which areas of the curriculum led to the most failures and therefore needed most attention, and which were the most likely subjects on which they would be questioned at each part of the examination. They therefore planned their revision so that they made sure they understood all the essential areas and more or less ignored the others that would have little bearing on the outcome of the examination. This group were the best organised; they met together frequently and exchanged views and regarded their performance in the examination as a collective exercise in which any useful hints obtained would be shared with others. This group hardly ever failed the examination and were sometimes mistakenly regarded as members of the 'deep thinking' group by the teachers.

## Relevance to Readers

Readers of 'Psychiatry Investigation' are more likely to come into the second and third groups that I describe above. Many of you will be extremely keen to learn, determined to advance knowledge, and ambitious to be published in the best possible journals. The rest of this editorial discusses what I would regard as the strategic group attitude towards research, or put more colloquially 'how to get the best possible return from the same degree of effort'. In making these suggestions I am not regarding the strategic thinkers amongst you as necessarily absent from the 'deep thinking' group, but until you are able to get to a certain status level it is very difficult for the 'deep thinking' group to have a major influence on science.

The first rule is to join an established research group. The days when single researchers like Mendel and Semmelweiss could change the world by working singly in a research area are now almost gone (They are not absolutely gone so I would not abandon this approach entirely). Nevertheless, because of the open nature of global science it is very rare for a single individual to pick on something which is entirely new and it is therefore best to align yourself with an established research group. For example in the September issue of 'Psychiatry Investigation' there was an overview of the Korean Longitudinal Study on Health and Ageing (KLoSHA).^1^ A large group such as this linked around a complex investigation is normally very successful in publication terms. As one of my colleagues in a well known medical journal once told me, 'if all the experts in the field contribute to an article it is bound to be widely cited because everyone will quote themselves'. Such papers tend to be popular with editors for this reason and almost have a guaranteed publication record. You will also notice that such papers often have many authors, and in the case of the KLoSHA study, you will note that there are seventeen authors, ten more than any other article in the September issue. Many people consider it to be unimportant to be one of seventeen authors and would much prefer this to be reduced to one. However the strategic thinker realises that the position of an author in a multi-authored paper is very important. According to the research attribution calculations of my employing institution, Imperial College, the first and last authors of a multi-author publication get a score of five in impact terms, the second author gets a score of four, the third and fourth authors get a score of three and two respectively with all other authors getting a score of one.^2^ The strategic thinker therefore tries very hard to put himself or herself in one of these better placed positions when the final author-ship is decided. The consequence of this tendency, the number of authors in highly cited publications has steadily increased.

## Change in Authorship of Journals

This is illustrated in Table 1 in which the number of authors in the January issue of the British Journal of Psychiatry over a forty year period is illustrated. You will note that the average number of authors per paper has increased dramatically in recent years (2.5 fold over the whole period) and this is not because there are fewer single or two-author publications being received; they are just more likely to be rejected. In giving these data I am not necessarily saying that multi-author investigations are good or bad; but can only conclude that it is a useful strategic exercise to get involved in such publications as they seem more likely to be published.

The second strategy is to pick on a new or rapidly expanding area of research. In Table 2 the impact factors of the top twenty psychiatric journals (calculated in 2006) are illustrated. This shows that four of the top eight psychiatric journals are in the related fields of biological psychiatry and neuropharmacology and general journals with a more straightforward clinical profile are relegated to a lower status. The good strategic thinker therefore identifies the growth areas early on (just as someone who invests in the stock market picks on a stock before it has become fashionable), and before long they will find themselves to be leaders in the field.

The third strategy is to go for short term gain. Some of my colleagues and joined large research groups thinking it will help their careers, but before long they find that the timescale of the studies concerned is so long that their chances of publication within the next five years are extremely remote. In studying subjects to research it is therefore always reasonable to ask 'when is the payoff?' The good strategic thinker can work this out fairly easily; a long term project may have many possibilities for ancillary papers or 'spin-offs' which allow publication before the final study is completed. The possibility of attaching yourself to a long term study in such a way that you can test a separate ancillary hypothesis in the short term is an ideal way of proceeding in this manner.

It may be felt that some of these suggestions are cynical and not likely to advance the cause of science significantly. I disagree, advancement in science depends on inspiration and luck but also depends on strategy. When Marie Curie decided to work on the investigation of new elements, believing that radioactivity was a common property of many of them, eventually leading to the discovery of radium and polonium, she came into the area with a cold and calculating attitude to the subject, realising that it had many risks but strategically it was likely to repay her with massive dividends. She did not just idly move into the territory; she planned it methodically and well. Everyone now recognises her as a brilliant scientist, but she was also a canny one and may not have achieved what she did without the use of the strategic approach I have outlined above.

## Figures and Tables

**TABLE 1:**
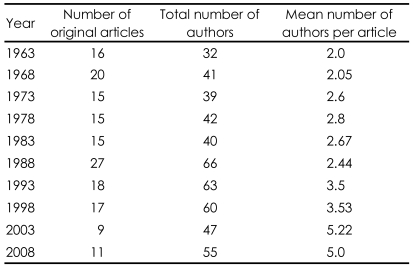
Quinquennial analysis of numbers of papers and authors in January issue of the British Journal of Psychiatry from 1963 to 2008

**TABLE 2:**
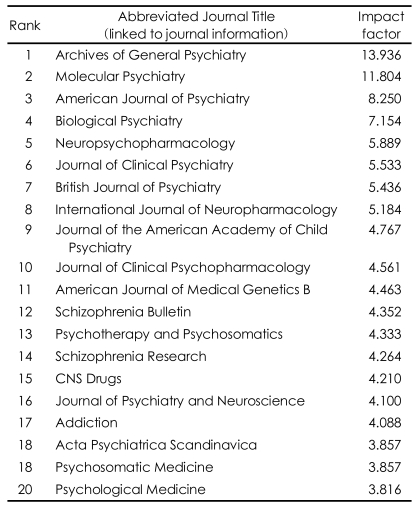
Impact factor of top 20 psychiatric journals in 2006 (from ISI Web of Knowledge http://wok.mimas.ac.uk/http://wok.mimas.ac.uk/)
